# Coagulopathy triggered autoimmunity: experimental antiphospholipid syndrome in factor V Leiden mice

**DOI:** 10.1186/1741-7015-11-92

**Published:** 2013-04-04

**Authors:** Aviva Katzav, Nikolaos C Grigoriadis, Tania Ebert, Olga Touloumi, Miri Blank, Chaim G Pick, Yehuda Shoenfeld, Joab Chapman

**Affiliations:** 1 Department of Neurology and Sagol Center for Neurosciences, Sheba Medical Center, Tel-Hashomer, 2 Sheba Rd, Ramat Gan, 52621, Israel; 2 Department of Physiology and Pharmacology, Sackler Faculty of Medicine, Tel Aviv University, 55 Haim Levanon St, Ramat Aviv, Tel Aviv, 69978, Israel; 3 Zabludowicz Center for Autoimmune Diseases, Sheba Medical Center, affiliated to Sackler Faculty of Medicine, Tel Aviv University, Tel-Hashomer, 2 Sheba Rd, Ramat Gan, 52621, Israel; 4 Department of Neurology, AHEPA University Hospital, Aristotle University of Thessaloniki, St. Kyriakidis 1, Thessaloniki, 54636, Greece; 5 Beer Yaakov-Ness Ziona Mental Health Center, 1 Haim Rd, Beer Yaakov, 70350, Israel; 6 Department of Anatomy and Anthropology, Sackler Faculty of Medicine, Tel Aviv University, 55 Haim Levanon St, Ramat Aviv, Tel Aviv, 69978, Israel; 7 Department of Neurology, Sackler Faculty of Medicine, Tel Aviv University, 55 Haim Levanon St, Ramat Aviv, Tel Aviv, 69978, Israel

**Keywords:** Autoimmunity, Coagulopathy, Antiphospholipid syndrome, Factor V leiden, Experimental antiphospholipid syndrome, Autoantibodies, Hyperactivity, Cognitive dysfunction, Neurodegeneration

## Abstract

**Background:**

We investigated interactions between genetically and autoimmune-mediated coagulopathies by inducing experimental antiphospholipid syndrome (eAPS) in mice carrying the factor V Leiden (FVL) mutation.

**Methods:**

eAPS was induced in heterozygous and homozygous FVL transgenic mice (C57BL/6 background) by immunization with β_2_-glycoprotein I (β_2_-GPI). Autoantibody levels were measured at 1 and 5 months post-immunization. Mice were tested at 4 months post-immunization for behavior and cognitive function in the staircase, elevated plus-maze, and swim T-maze tests. Brains were removed and analyzed by immunohistochemistry for inflammatory markers and neurodegenerative processes.

**Results:**

A single immunization with β_2_-GPI induced significantly higher and longer-lasting immune responses, and this was dependent on the number of FVL alleles. At 1 and 5 months post-immunization, levels of antibodies rose from 1.17 ± 0.07 to 1.62 ± 0.17 (optical density units; ODU) in homozygous FVL mice, compared with stable levels of 0.59 ± 0.17 and 0.48 ± 0.16 ODU in heterozygous FVL mice and a drop from 1.62 ± 0.21 to 0.61 ± 0.13 ODU in wild-type mice. Behavioral and cognitive clinical features of eAPS were also correlated with FVL allele load, as assessed by the elevated plus-maze (altered anxiety), staircase (hyperactivity and higher exploration), and swim T-maze (impaired learning) tests. Histological studies identified significant neurodegenerative changes in both grey and white matter in the eAPS-FVL brains. In spite of the potential interaction of two prothrombotic disease states, there were no ischemic lesions seen in this group.

**Conclusions:**

The results indicate that genetically mediated coagulopathies increase the risk of developing coagulation-targeted autoimmune responses, and suggest the importance of antibody-mediated neurodegenerative processes in the brain in APS.

## Background

Blood coagulation factor V (FV) is a pivotal protein in hemostasis, playing a crucial role in both the procoagulant and anticoagulant pathways [1,2]. FV serves as a cofactor of factor Xa in the prothrombinase complex that converts prothrombin to active thrombin. FV is inactivated by activated protein C (APC). FV Leiden (FVL) is a genetically acquired trait that can result in a thrombophilic (hyprcoaguable) state, resulting in the phenomenon of APC resistance. The FVL allele is present in about 5% of Caucasian populations (European, Jewish, Israeli Arab, and South Asian (Indian) populations) and is virtually absent in Africans and East Asian populations [3,4]. FVL is the most common cause of inherited thrombosis, accounting for 40-50% of cases [1].

Antiphospholipid syndrome (APS) is the most important acquired thrombophilic defect. APS is characterized by the presence of anti-phospholipid (aPL) antibodies and by occurrence of clinical features including repeated spontaneous abortions, thrombocytopenia, systemic thrombosis, and central nervous system (CNS) dysfunction. aPL antibodies are a heterogeneous group of circulating autoantibodies directed against negatively charged phospholipids and phospholipid-binding proteins, such as β_2_-glycoprotein I (β_2_-GPI) and prothrombin [5]. Because thrombosis does not occur in all patients with aPL antibodies, it is likely that additional factor(s) determine the clinical presentations of APS. An association of FVL and APS has been reported previously [6-8], and this coexistence of APS and FVL has been hypothesized to occur by chance and to increase the risk of thrombosis [9]. aPL antibodies have been found to inhibit APC anticoagulant function and cause acquired APC resistance [10-15]. However, there are few epidemiological data available about the prevalence of aPL antibodies in subjects with and those without FVL.

We have developed a consistent and reproducible animal model of the CNS effects of APS [16,17]. This model is induced in female mice by immunization with the autoantigen β_2_-GPI. Following a single immunization, these mice develop sustained high levels of autoantibodies to phospholipids and over a period of 4–5 months, the mice display significant behavioral changes and cognitive deficits. A knock-in transgenic model of FVL has been developed by Ginsburg et al. [18]; these mice are prothrombotic but otherwise phenotypically normal.

In the present study, we examined the interaction of APS with FVL using our mouse model to investigate whether there is an increased risk of thrombosis and exacerbation of the experimental APS (eAPS) phenotype. The results indicated that there is a significant enhancement of both APS linked antibodies and brain dysfunction but unexpectedly, without significant ischemic pathology.

## Methods

### Ethics approval

The Tel Aviv University Animal Welfare Committee approved all procedures.

### Mice

The transgenic mice used in this study (kindly provided by Professor David Ginsburg, University of Michigan, Ann Arbor, MI, USA) carry the ortholog of the human FVL mutation previously generated by a knock-in of the R504Q mutation into the endogenous murine factor V locus by homologous recombination [18]. These mice were back-crossed to C57BL/6 mice for more than seven generations. Genotyping of the offspring for the FVL transgene was performed by PCR with previously described primers, using DNA obtained from tail biopsies taken post-weaning. The mice were raised under standard conditions, 23 ± 1°C, 12 -hour light cycle (0700 to 1900 hours) with *ad libitum* access to food and water.

### Preparation of β_2_-GPI

Human plasma was used as a source of β_2_-GPI by the method of Polz et al. [19]. In brief, serum proteins were precipitated by perchloric acid, and the remaining supernatant was adjusted to pH 8 by adding a saturated Na_2_CO_3_ solution. This fraction was dialysed exhaustively against 0.03 M NaCI pH 8 at 4°C, and further purified by affinity chromatography on heparin column (HiTrap Heparin HP, GE Healthcare Life Sciences, UK). Fractions containing β_2_-GPI were eluted with 0.35 mol/l NaCl, then separated by protein electrophoresis and visualized with silver stain. Fractions used for immunization contained a major band that was shown by western blotting to cross-react with a commercial antibody to β_2_-GPI (anti-ApoH; CSL Behring, Marburg, Germany) [20].

### Induction of experimental antiphospholipid syndrome

Mice heterozygous (FVL^Q/+^) and homozygous (FVL^Q/Q^) for the FVL transgene were immunized by a single intradermal injection with 10 μg of β_2_-GPI emulsified in complete Freund’s adjuvant (CFA). The control group comprised FVL^Q/+^ mice immunized similarly with CFA. C57BL/6 mice were immunized with either β_2_-GPI in CFA or CFA alone.

### Study design

In the first experiment, both female and male FVL^Q/+^ mice were divided into two groups of fifteen each. Each group included seven to eight mice immunized with β_2_-GPI (eAPS mice), and seven to eight mice immunized with CFA (adjuvant-immunized controls). In the second experiment, female FVL^Q/Q^ mice (n = 7) were immunized with β_2_-GPI, and female FVL^Q/+^ mice (n = 8) were immunized with CFA. Mice were immunized at 3 to 4 months of age, and behavioral assessment was started 4 months later with the staircase test, followed by the elevated plus-maze test and the swim T-maze test on the following sequential days.

### Serological evaluation

For serological evaluation, blood samples were collected from all the mice described above at 1 and 5 months after immunization. Autoantibody measurements were additionally performed in naive FVL^Q/+^ mice (n = 7), and naive C57BL/6 mice (n = 9). Autoantibody levels in these experiments were also compared with those in C57BL/6 mice with experimental APS induction (n = 10 and n = 11 for C57/B6-APS and C57/B6-control mice, respectively).

Blood samples were collected by retro-orbital sinus puncture as soon as the mice completed their behavioral and cognitive assessment. The sera were separated by centrifugation and stored at −70°C until assayed. The sera were tested by standard ELISA for the presence of autoantibodies as previously described [21], using serum-dependent (β_2_-GPI) and serum-independent antibodies to cardiolipin (CL) and phosphatidylserine, and antibodies to β_2_-GPI and double-stranded DNA.

### Staircase test

The staircase apparatus consisted of a polyvinylchloride (PVC) enclosure with five identical steps, 75 × 100 × 25 mm, on top of each other. The inner height of the walls above the level of the stairs was consistent (125 mm) along the whole length of the staircase. The box was placed in a room with constant lighting and isolated from external noise. Each mouse was tested individually. The animal was placed on the floor of the staircase with its back to the staircase. The number of stairs climbed and the number of rears during a 3-minute period were recorded. Climbing was defined as each stair on which the mouse placed all four paws; rearing was defined as each instance the mouse rose on hind legs (to sniff the air), either on a stair or leaning against the wall. The number of stairs descended was not taken into account. Before each test, the box was cleaned with a diluted alcohol solution to eliminate smells.

### Swim T-maze

A three-arm, walled T-maze,constructed of white Plexiglas (600 mm along the stem, 800 mm side at the T-intersection, 400 mm high, with passages 100 mm wide), was situated in one corner of a brightly lit behavioral-testing room separate from the colony. The T-maze was refilled daily with 145 mm of water at 2°C so that a platform (140 mm high, 300 mm^2^ in size), rising from the floor of the maze, was submerged 5 mm below the water line. One day prior to initial training, mice were placed in the maze and allowed to swim for 60 seconds with no platform present. The platform was then inserted in a standardized position 80 mm from the end of a goal arm, and each mouse was placed directly on the platform for 30 seconds. Finally, each mouse was placed at the far end of the stem and allowed to locate the submerged goal-arm platform. On each of four consecutive training days, a forced-choice alternation paradigm required each subject to perform eight replications of a paired forced-choice/free-choice trial sequence. With either the left or right goal arm blocked with a guillotine door, each subject was placed in the far end of the stem, and allowed to ascend the submerged platform located in the goal arm opposite the blocked arm. The animal remained atop the platform for 15 seconds at the conclusion of this forced choice trial. The animal was then removed by the tail and again placed at the end of the stem, while simultaneously, the guillotine door was removed and the platform moved to the opposite goal arm; that is, the one previously blocked. The latency period for the mouse to reach the platform and the number of correct choices, defined as entry into the goal arm with a platform prior to entry into the goal arm without a platform or re-entry into the stem, were measured during these free-choice trials. Each subject was again allowed to remain on the platform for 15 seconds and was then replaced in the home cage. The goal arm designated for forced choice (right or left hand) alternated from trial to trial over the eight trials of a daily session, from animal to animal over the course of a single day’s testing, and from day to day in terms of trial 1. Subjects who failed to locate the platform within 1 minute were assigned a latency of 60 seconds, lifted from the water by the tail, and placed atop the platform. The results were analyzed as the percentage of correct choices, using repeated-measures ANOVA.

### Elevated plus-maze test

The elevated plus maze was made from polyvinylchloride, and built in the shape of a plus sign, with two open (white) arms (340 × 75 × 10 mm) and two closed (black) arms (340 × 75 × 175 mm) opposite each other. The center of the four arms comprised the middle square (75 × 75 mm). The maze was elevated 510 mm above ground level. Each mouse was placed separately in the centre of the maze, facing an open arm, and allowed to explore the apparatus freely for 5 minutes. Parameters measured included the number of entries into the closed and open arms (an index of motor function), and the length of time spent in the closed and open arms. An entry was counted only after the mouse entered the arm with four paws. Before each test, the box was cleaned with a diluted alcohol solution to eliminate smells. The percentage of entries into the open arms out of the total number of arm entries and the percentage of time spent in the open arms, which are all accepted measures of anxiety levels, were further calculated.

### Histological studies

Mice were anesthetized by intraperitoneal injection of ketamine (100 mg/kg) and xylazine (20 mg/kg) and underwent transcardiac perfusion with phosphate buffer saline followed by perfusion with 4% paraformaldehyde in PBS. Brain tissue was collected, fixed in 4% paraformaldehyde and embedded in paraffin wax. Coronal sections 6 μm thick were cut, mounted, and stained with hematoxylin and eosin (H&E), Luxol Fast Blue (LFB), and Bielchowsky (BLS) stains to identify histological details and the density of myelin and axons, and the sections were specifically examined to evaluate ischemic pathology such as micro-infarcts.

### Immunohistochemistry

Paraffin wax-embedded sections were dewaxed and rehydrated in xylene and alcohol solutions, then rinsed with PBS. Citrate buffer was used for antigen retrieval, and endogenous peroxidase was blocked with 3% H_2_O_2_ in methanol. After incubation of the sections in blocking buffer (Foetal bovine serum, FBS) they were treated with primary antibodies against glial acidic fibrillary protein (GFAP; Dako, Glostrup, Denmark), MAC3, B220 (both BD Biosciences, Inc., San Jose, CA, USA), CD3 (Neomarkers Inc., Fremont, CA, USA), vascular endothelial growth factor (VEGF; Spring Bioscience Corp., Pleasanton, CA, USA), for the detection of astrocytes, macrophage/microglia, B cells, T cells, and VEGF, respectively (dilutions: 1;500, 1:100, 1:100, 1:150, 1:100, respectively). Immunoreactivity was visualized with a commercial system (EnVision HRP; Dako) and sheep anti–rat antibody (AbD Serotec, Raleigh, NC, USA). DAB (Sigma Chemical Co., St Louis, MO, USA) was used as chromogen. Counterstaining was performed with hematoxylin.

### Immunofluorescence staining

Immunofluorescence staining for Iba1 (rabbit polyclonal antibody, Wako, Osaka, Japan) was used to identify microglial and macrophage populations, and was performed using the same protocol as described above with the appropriate secondary antibody (goat anti-rabbit IgG conjugated to fluorescein isothiocyanate (AlexaFluor 488). Slides were mounted with DAPI (Invitrogen Corp., Carlsbad, CA, USA).

### *In vitro* immunohistological staining

We investigated which specific brain structures the aPL antibodies bind to, using brain sections of normal mice immunostained with pooled serum from FVL-eAPS and control mice. Normal brain sections were stained with pooled serum (diluted 1:200) overnight at 4°C and then with the secondary antibody (alkaline phosphatase conjugated anti-mouse IgG). Bound antibody was detected by development with Fast Red substrate (Sigma Chemical Co.) for 10 minutes, after which the sections were mounted with glycerol.

### Pathological evaluation

Sections were examined using fluorescence and optical microscopy (Axioplan-2; Carl Zeiss, Jena, German) with the aid of a CCD camera (DS-5Mc; Nikon, Tokyo, Japan) by two independent observers blinded to the experimental groups. The evaluation was performed for the whole brain, using the Paxinos and Franklin (2004) stereotaxic coordinates (ranging from bregma 2.22 to bregma −6.36) [22]. On average, 20 optical fields per slice and three slides per each group were examined under × 20 or × 40 magnification. Measurements were performed with ImageJ software (version 1.43; http://rsb.info.nih.gov/ij/), and data are presented as positive cells per mm^2^ for MAC3, CD3, B22O, VEGF, and GFAP. Additional evaluation for astrocytic and microglial activation was performed as the ratio of tissue area positive for GFAP/Iba1 per mm^2^ of total area studied. The density of myelin and axons was evaluated with Image J software, using a range of 0.05 to 3.05 OD units, and measurements were performed using a Rodbard function.

### Statistical analysis

Levels of antibodies and scores on the staircase and plus-maze tests were compared using one-way ANOVA followed by least squares difference *post hoc* tests. Performance on the swim T-maze was analyzed by means of repeated-measures ANOVA. Most statistical tests were performed using the SPSS software package for PC (SPSS Inc., Chicago, IL, USA). Statistical analysis of histological data was performed using GraphPad Prism software (version 5.0, GraphPad Software, La Jolla, CA, USA). The normality was tested using the Shapiro-Wilk and Kolmogorov-Smirnov tests. Non-parametric data were analyzed using the equivalent Kruskal-Wallis test followed by Dunn’s *post hoc* multiple-comparison test. Values of all scale data are expressed as mean ± SE. All determinations were made with 95% confidence interval and were considered significant at *P* < 0.05.

## Results

### Antiphospholipid autoantibody levels are increased in eAPS-FVL mic*e*

**Figure 1:**
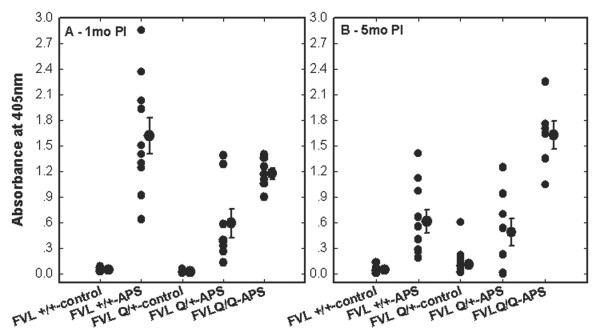
**Autoantibody levels in sera of factor V Leiden (FVL) and C57/B6 mice.** Antiphospholipid syndrome (APS) was induced in female mice by immunization with β_2_-glycoprotein I (β_2_-GPI), whereas controls were immunized with adjuvant (complete Freund’s adjuvant, CFA) alone. Anti-cardiolipin β2-GPI-dependent (anti-CL(β_2_-GPI)) antibodies were measured in APS (FVL^+/+^-APS, n = 10), control FVL^+/+^ C57BL/6 background (FVL^+/+^-control, n = 11), FVL heterozygous APS (FVL^Q/+^-APS, n = 8), FVL homozygous APS (FVL^Q/Q^-APS n = 7),and FVL heterozygous control (FVL-control, n = 15) mice. Titers were measured at **(A)** 1 month post-immunization (1mo PI) and **(B)** 5 months post-immunization (5mo PI). The levels of antibody represent individual and mean ± SE absorbance values for ELISA. The FVL mice, and especially the FVL^Q/Q^-APS group, developed significantly higher levels of anti-CL(β_2_-GPI) antibodies at 5mo PI compared with the control background mice, in which the levels of antibodies dropped over time.

We compared the effect of APS induction by immunization with β_2_-GPI on the levels of autoantibodies in female mice that were heterozygous FVL^Q/+^, homozygous FVL^Q/Q^, and FVL^+/+^ C57/B6 background naive mice (Figure 1). At 1 month after APS induction, high levels of β_2_-GPI-dependent anti-CL (anti-CL(β_2_-GPI)) antibodies were found in all APS-immunized mice compared with adjuvant-immunized (control) naïve and FVL mice (*P* < 0.001 for the effect of immunization by ANOVA) (Figure 1A). The level of anti-CL(β_2_-GPI) was significantly higher in FVL^+/+^-APS mice compared with FVL^Q/+^-APS and FVL^Q/Q^-APS mice (*P* < 0.045, ANOVA) and in FVL^Q/Q^-APS mice compared with FVL^Q/+^-APS mice (P = 0.013, ANOVA). Four months later (5 months after the single immunization), the level of anti-CL(β_2_-GPI) had dropped significantly in the FVL^+/+^-APS mice whereas it had risen in the FVL^Q/Q^-APS mice (*P* < 0.001 and *P* = 0.007, ANOVA, respectively, compared with the level at 1 month after APS induction) (Figure 1B). The anti-CL(β_2_-GPI) levels in FVL^Q/+^-APS mice remained high at 5 months after immunization, and were similar to the levels at 1 month (*P* = 0.6, ANOVA). At 5 months after immunization, the anti-CL(β_2_-GPI) levels in the FVL^Q/Q^-APS mice were significantly higher compared with both the FVL^+/+^-APS and FVL^Q/+^-APS mice (*P* < 0.001, ANOVA).

The effect of sex on autoantibodies in FVL^Q/+^ APS mice was also examined. One month after immunization, both female and male FVL^Q/+^ APS mice developed high levels of anti-CL(β_2_-GPI) (0.59 ± 0.17 and 0.30 ± 0.14 ODU, respectively) compared with their FVL adjuvant-immunized controls (0.02 ± 0.002 and 0.02 ± 0.003 ODU, respectively), and there was no significant difference between the sexes (*P* = 0.001 for immunization effect and *P* = 0.28 for sex effect, ANOVA). However, 4 months later (that is, 5 months post-immunization), the level of anti-CL(β_2_-GPI) was significantly higher in the female(0.48 ± 0.16) than in the male (0.21 ± 0.08) FVL-APS mice, (*P* = 0.037 for sex effect, ANOVA).

### Functional brain changes in FVL-APS mice

**Figure 2:**
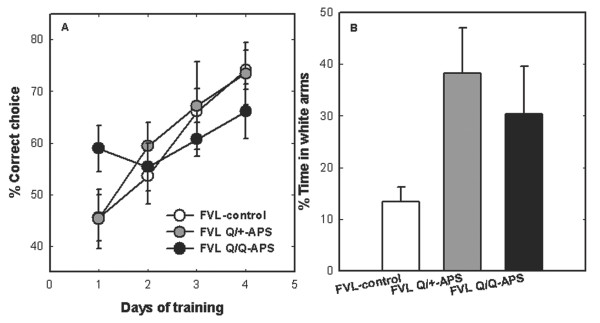
**Effects of antiphospholipid syndrome (APS) induction on behavior and cognition in factor V Leiden (FVL) mice. (A)** Cognitive function in a swim T-maze alternation test. The results are presents as the mean ± SE proportion (%) of correct choices in eight trials/day over 4 consecutive days. There was significantly impaired learning in the FVL^Q/Q^-APS mice (*P* = 0.026 for the interaction days × group, by repeated-measures ANOVA). **(B)** Anxiety-related behavior in an elevated plus-maze test. The results are presented as mean ± SE time (%) spent in the white (open) arms. Both the FVL^Q/+^-APS and FVL^Q/Q^-APS mice spent significantly more time in the white arms compared with FVL-control mice (*P* < 0.031 by ANOVA), indicating altered levels of anxiety induced by APS in FVL mice. Cumulative data from two independent experiments (FVL^Q/+^-control (FVL-control), n = 15; FVL^Q/+^-APS, n = 8; FVL^Q/Q^-APS, n = 7).

**Figure 3:**
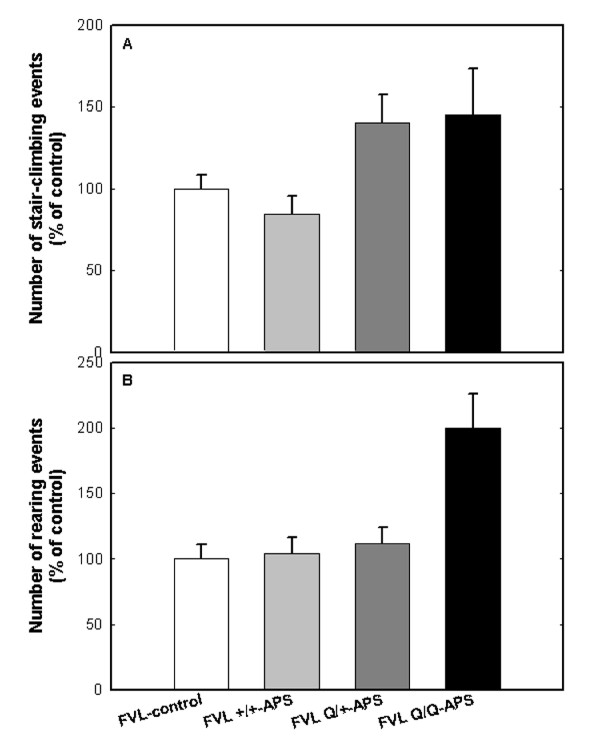
**Hyperactivity in the staircase test linked to gene dosage and autoantibody levels in experimental antiphospholipid syndrome factor V Leiden (eAPS-FVL) mice.** Behavioral measurements in the staircase test included activity and exploration. The results are presented as mean number of events (percentage of the control group (FVL-control)). **(A)** For the activity parameter (stair-climbing), FVL^Q/+^-APS and FVL^Q/Q^-APS mice were hyperactive compared with both their FVL^Q/+^-control (FVL-control) group and the FVL^+/+^-APS mice. **(B)** For the exploratory parameter (rearing), the FVL^Q/Q^-APS mice had significantly higher levels of exploration compared with the FVL^Q/+^-controls (FVL-control), whereas no significant difference was found between the FVL^Q/+^-APS mice and the controls (*P* = 0.006 and *P* = 0.29, respectively). Cumulative data from two independent experiments (FVL^Q/+^-control, n = 15; FVL^Q/+^-APS, n = 8; FVL^Q/Q^-APS, n = 7; FVL^+/+^-APS, n = 10).

Cognitive function in the swim T-maze test, behavioral features of anxiety in the elevated plus-maze test, and activity/exploration in the staircase test were measured 4 months after APS induction in female FVL transgenic mice (Figure 2, Figure 3). The results of the swim T-maze are presented as the mean percentage of correct choices during eight trials per day over 4 consecutive days (Figure 2A). There was significantly impaired learning in the FVL^Q/Q^-APS mice (*P* = 0.026 for the interaction days × group by repeated-measures ANOVA) but no significant difference in learning (improvement over time) between the FVL^Q/+^-APS and FVL^Q/+^-control (FVL-control) mice.

The results of the elevated plus-maze test are presented as the mean percentage time spent in the white (open) arms (Figure 2B). Both the FVL^Q/+^-APS and FVL^Q/Q^-APS mice spent significantly more time in the white arms compared with the FVL-CFA controls (*P* < 0.031 by ANOVA), indicating altered levels of anxiety induced by APS in FVL mice.

Behavioral measurements in the staircase test included activity and exploration (Figure 3). The results are presented as the mean number of events relative to the appropriate control group of wild-type (C57BL/6) or FVL mice (= 100%). In the activity measure (stair-climbing; Figure 3A), FVL^Q/+^-APS and FVL^Q/Q^-APS mice were hyperactive compared with their FVL^Q/+^ controls (FVL control, *P* < 0.035 for the effect of immunization) and with FVL^+/+^-APS mice, which were hypoactive relative to their wild-type controls. In the exploratory measure (rearing; Figure 3B), the FVL^Q/Q^-APS mice had significantly higher levels of exploration compared with the FVL^Q/+^-CFA controls, whereas no significant difference was found between the FVL^Q/+^-APS and the FVL^Q/+^-CFA controls (*P* < 0.001 and *P* = 0.26, respectively).

### Ischemic events

Only two clinically overt stroke incidents occurred, which were both in the first group of animals examined. These were a right middle cerebral artery (MCA) ischemic event and a venous thrombosis, which occurred spontaneously in FVL^Q/+^ mice 1 week after immunization with β_2_-GPI. The strokes were identified when the animals developed severe motor signs and were hemiplegic or obtunded. The diagnosis was verified by macroscopic examination and magnetic resonance imaging scans of the brains. The other mice did not display any focal motor weakness throughout the study period.

### Histology studies show mainly neurodegenerative changes

**Figure 4:**
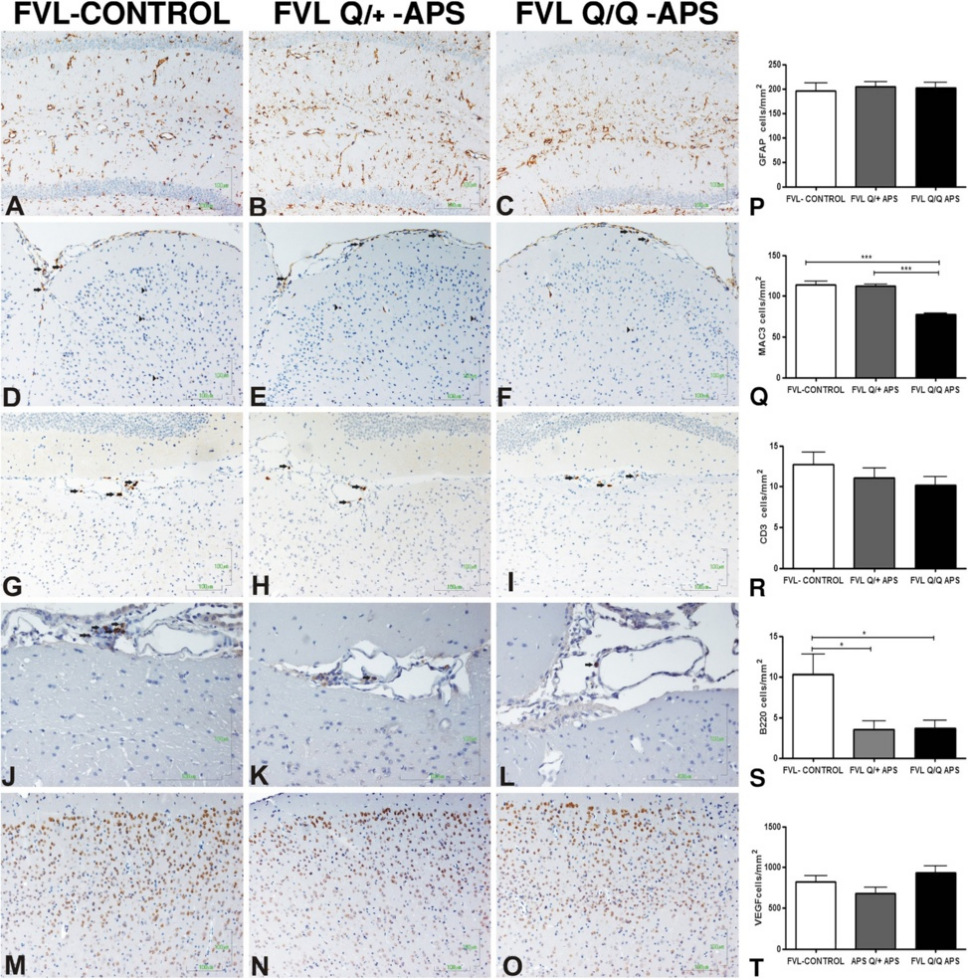
**Immunohistochemical staining for inflammatory and vascular markers in factor V Leiden (FVL) mice.** Representative immunohistochemical staining images from the three groups: adjuvant-immunized FVL control (FVL-control), experimental antiphospholipid syndrome (eAPS), heterozygous FVL (FVL^Q/+^-APS) and eAPS homozygous FVL (FVL^Q/Q^-APS) mice. Quantification data for each marker are also presented. **(A–C,P)** Glial fibrillary acidic protein (GFAP)-positive immunoreactions with similar expression in the area of the hippocampus (original magnification × 20). **(D–F,Q)** MAC3-positive cells (macrophages) in the meninges (black arrows) and in the parenchyma of the cortex (black arrowheads; original magnification × 20). **(G–I,R)** CD3-positive cells (T cells, black arrows; original magnification × 20). **(J–L,S)** Infiltrates with increased expression of B220-positive cells (B cells) in the control FVL group compared with the APS FVL^Q/+^ and APS FVL^Q/Q^ groups (black arrows; original magnification × 40). **(M–O,T)** Representative images of vascular endothelial growth factor (VEGF) staining, with similar expression in the area of the cortex (original magnification × 20).

The histological studies performed on FVL and control mice brains at 5 months post-immunization were aimed at assessing ischemic, inflammatory, and neurodegenerative changes. Examination of blood vessels and of brain structures for the presence of overt ischemic lesions was performed using immunohistochemistry staining for VEGF, and histochemical staining with H&E and white-matter LFB. There was no gross pathology compatible with strokes in any brain structures including the hippocampus, (Figure 4) and there was no specific pathology in blood vessels (Figure 4M-O,T). Inflammatory markers were assessed by staining for macrophages (MAC3), B- ells (B220) and T cells (CD3); quantitative assessments of these stains are presented in Figure 4. There was a significant reduction in the B-cell marker B220 in eAPS mice with FVL backgrounds (both FVL^Q/+^ and FVL^Q/Q^) compared with adjuvant-immunized heterozygous FVL^Q/+^ control mice (Figure 4J-L,S). A reduction in the macrophage marker MAC3 was also found in the FVL^Q/Q^-APS mice compared with both the eAPS and the adjuvant-immunized FVL^Q/+^ mice (Figure 4D-F,Q). There was a non-significant similar trend for fewer T cells in the eAPS-FVL mice compared with FVL^Q/+^ adjuvant-immunized controls (Figure 4G-I,R).

**Figure 5:**
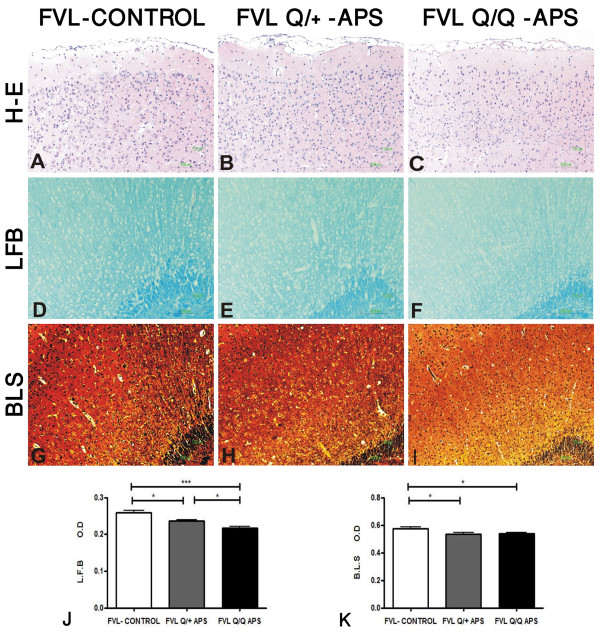
**Demyelination and axonal injury in the experimental antiphospholipid syndrome factor V Leiden (eAPS-FVL) groups.** Representative images from the area of the cortex in three groups: adjuvant immunized FVL control (FVL-control), heterozygous eAPS FVL (FVL^Q/+^-APS) and eAPS homozygous FVL (FVL^Q/Q^-APS) mice. Staining with **(A–C)** hematoxylin and eosin (H&E), showing mild infiltration in the meninges; **(D–F)** Luxol fast blue (LFB) to measure myelin density; and **(G–I)** Bielchowsky (BLS) to measure axon density. Quantitative data from all brain slices stained with **(J)** LFB and **(K)** BLS confirmed significant FVL^Q^-gen-dose-dependent white-matter neurodegenerative changes in the eAPS mice compared with the adjuvant-immunized controls. The axonal BLS stain showed that both eAPS-FVL groups had similar significant reductions in staining density, compatible with neurodegeneration. Original magnification × 20.

**Figure 6:**
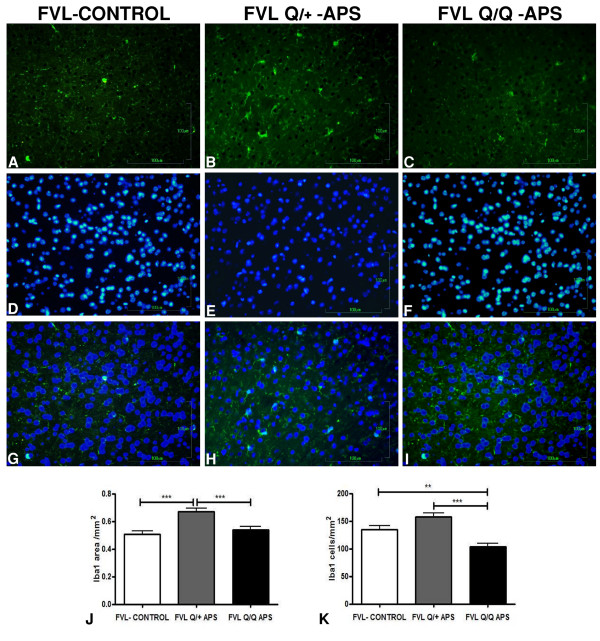
**Changes in activated microglia in factor V Leiden antiphospholipid syndrome (FVL-APS) mice seen by Iba1 immunoreactivity.** Representative images stained with **(A–C)** Iba1, **(D–F)** DAPI**. (G–I)** Double-stained pictures were merged. **(J,K)** Iba1 immunoreactivity measurements (expressed as area/mm^2^) showed **(J)** a significant increase in the FVL^Q/+^-APS group compared with the FVL adjuvant-immunized control and FVL^Q/Q^-APS groups (*P* < 0.001), **(K)** and a significant decrease in the FVL^Q/Q^-APS group compared with the FVL^Q/+^-APS and the FVL-control groups (*P* < 0.01).

The most striking differences between the groups were found for measures of neurodegeneration as shown with LFB and BLS staining, which indicated demyelination and axonal loss in the eAPS-FVL groups compared with the control adjuvant-immunized FVL^Q/+^ group. Figure 5 displays representative hippocampal slices showing these gene-dose-dependent changes in both pathological measures, which were also detected throughout the cortical areas. Quantitative data from all brain slices stained with LFB and BLS confirmed significant FVL^Q^ gene-dose-dependent white-matter neurodegenerative changes in the eAPS mice compared with adjuvant-immunized controls (Figure 5J,K). In the axonal BLS-stained sections, both eAPS-FVL groups had similar significant reductions in density of staining, compatible with neurodegeneration (Figure 5G-I,K), although there was no evidence of ongoing acute axonal degeneration (spheroids, ovoids) at the time of brain-tissue sampling. In contrast to the axon and myelin measures, there was no difference between the groups in the number or intensity of glia that stained for GFAP (Figure 4A-C,Q). Changes in activated microglia were seen (Figure 6) using Iba1 immunoreactivity (expressed as area/mm^2^) which showed a significant increase in the FVL^Q/+^-APS group compared with the FVL adjuvant-immunized control group (Figure 6J) and the FVL^Q/Q^-APS group (*P* < 0.001). The Iba1 measurements (cells/mm^2^) showed a significant increase in the FVL^Q/+^-APS group compared with the FVL^Q/Q^-APS group (*P* < 0.001) (Figure 6K). The FVL^Q/Q^-APS group also displayed a marked reduction in Iba1 staining compared with the FVL-control group (*P* < 0.01).

### *In vitro* staining with pooled mouse IgG

**Figure 7:**
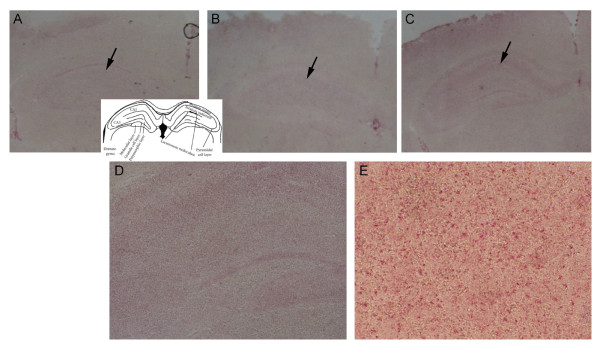
**Specific binding of factor V Leiden experimental antiphospholipid syndrome (FVL-eAPS) mouse sera to normal brain.** Representative slides of normal brain stained with pooled serum from adjuvant control and eAPS-FVL mice. Low-magnification microscopy of brain slices stained with **(A)** pooled adjuvant control sera, **(B)** FVL^Q/+^-APS sera, and **(C)** FVL^Q/Q^-APS sera showed significant staining of white-matter areas of the hippocampus (black arrows, original magnification × 2). **(A, insert)** Hippocampal layers. **(D,E)** Higher-magnification pictures showed significant staining of cells by pooled eAPS-FVL^Q/Q^ sera, compatible with the presence of interneurons outside the main pyramidal cell layers of the cornu ammonis (CA) 1 and 3, and were localized in the stratum radiatum area. Original magnification (D) × 10, (E) × 40.

To examine whether the staining pattern was the result of the antigenic specificity of the mouse antibodies, normal mouse brains were stained with pooled serum from eAPS-FVL mice (representative slides are presented in Figure 7). Low-magnification microscopy of brain slices stained with pooled eAPS sera showed significant staining of white-matter areas in the hippocampus. This binding was more pronounced in the homozygous FVL^Q/Q^-APS mice (Figure 7C) than in the FVL^Q/+^-APS mice (Figure 7B), whereas no such staining was seen in the brain slices stained with pooled sera from adjuvant-immunized control mice (Figure 7A). Higher-magnification images (Figures 7D and E) showed significant staining by pooled eAPS-FVL^Q/Q^ sera of cells compatible with interneurons outside the main pyramidal cell layers of the cornu ammonis 1 and 3, and these were localized to the stratum radiatum area. No such staining was seen in similar areas stained with pooled control sera.

## Discussion

In the present study, the main findings were an increase in aPL antibody levels and a number of behavioral/cognitive dysfunction and neurodegenerative changes associated with these autoantibodies in the FVL eAPS mice. These effects were linked to gene dosage, and were thus significantly more pronounced in homozygous FVL^Q/Q^ than in heterozygous FVL^Q/+^ mice. The serological and behavioral effects found in the FVL mice in this study are in line with results of previous studies using other mouse strains in which eAPS was induced by immunization with β_2_-GPI [17,23,24] or immunization with a pathogenic autoantibody [25].

The pathogenesis of aPL antibody-mediated brain damage is complex, and may include thrombosis, inflammation, or direct binding of antibodies to the brain. The results of the present study do not support the presence of either thrombosis or inflammation in the FVL eAPS brain. This is in line with previous studies in the eAPS model in various wild-type strains of mice, including C57BL/6 [26,27]. It is also compatible with findings in human APS, as a significant number of patients with neurological features do not have imaging or clinical findings supporting thrombosis or brain inflammation [28,29].

In view of the lack of thrombosis or inflammation, the most significant pathogenic factor explaining the interaction of FVL and APS are the very high levels of aPL antibodies. The levels of these antibodies in β_2_-GPI-immunized wild-type C57BL/6 mice are known to decline significantly over time [17,23], which is in contrast to the continuing increase seen in the homozygous FVL^Q/Q^ mice and in the stable levels found in the heterozygous FVL^Q/+^ mice in the current study. It should be stressed that all β_2_-GPI-immunized eAPS mice in the present study received only one immunization, and that the response in FVL mice is unusually strong and prolonged compared to the response in C57BL/6 mice [23]. These very high levels of aPL antibodies correlate with the behavioral and cognitive deficits, which are not found in wild-type C57BL/6 eAPS mice [23], and indeed induction of APS in wild-type C57BL/6 mice causes hypoactivity rather than the hyperactivity seen in the FVL mice. These findings suggest that the brain is affected by antibodies in a manner not mediated primarily through thrombosis or inflammation. A major potential pathogenic mechanism is the direct binding of aPL antibodies to brain cells such as neurons, and subsequent specific dysfunction of these cells. This mechanism is well established in diseases such as myasthenia gravis and Lambert-Eaton myasthenic syndrome [30], and in CNS autoimmune diseases such as the anti-potassium channel antibody spectrum and paraneoplastic autoimmune diseases [31,32]. We have shown previously that APS [33] and eAPS sera [24] do bind to neurons in specific limbic areas of the normal mouse brain. In the present study, we found specific binding of IgG from FVL-APS mice to normal brain, mainly to neurons in the white-matter area of the hippocampus and cortex. This binding was more pronounced in the FVL^Q/Q^-APS than the FVL^Q/+^-APS mice, and was not seen with sera of the adjuvant-immunized control FVL mice. This supports our suggestion that binding of IgG directly to the brain may be a major pathogenic mechanism in these mice.

The potential effects of direct binding of antibodies to the mouse brain are neuronal dysfunction and death, which would essentially lead to a neurodegenerative process. Neurodegeneration is indeed supported by the histological results in the present study, with both neuronal cell-body loss and axon loss seen in the FVL eAPS mice in a gene-dose/antibody-level-dependent fashion. The increased microglial activation in FVL eAPS mice is probably best explained as a secondary response to neurodegenerative changes, as this was not accompanied by changes in astrocytes or by overt inflammation with changes in astrocytes, macrophages, or lymphocytes. This finding again suggests that neurodegenerative processes in human APS may well be explained by high aPL antibody levels.

A trend towards a, FVL gene-dose-dependent exaggerated response to immunization was found in the eAPS mice. The finding that the wild-type FVL^+/+^ mice had the highest initial levels of aPL antibodies at 1 month indicates that expression of the gene does not have an immediate stimulatory effect on the immune system. It was at the later time point (5 months) that aPL antibody levels were increased in the FVL^Q/+^ mice, and this effect was dramatically more pronounced in the FVL^Q/Q^ mice. The most reasonable explanation for this late and prolonged elevation of aPL antibody levels in FVL^Q/Q^ mice is that of an ongoing process, such as the chronic exposure of the immune system to activated components of the clotting system, which include β_2_-GPI. This is in line with our recent publication describing spontaneous development of specific pathogenic aPL (β_2_GPI-dependent) autoantibodies in genetically hypercoagulation-prone FVL mice immunized with adjuvant alone [34]. Interestingly, adjuvant-immunized FVL mice exhibited hyperactivity behavior compared with non-immunized FVL mice, which correlated with the autoantibody level [34]. These results are in line with the hyperactivity behavior displayed in an experimental APS model induced in naive strains [16,17,23,27], in a transgenic mouse model of Alzheimer’s disease [35], and in transgenic FVL mice (the present study).

We therefore propose the following hypothesis for the mechanism of specific generation of pathogenic aPL antibodies in FVL mice. Autoantibodies to coagulation factors and associated proteins are commonly described in patients with APS [36,37], and β_2_-GPI itself is intimatelyassociated with the coagulation process. In a situation such as FVL, in which there is chronic uncontrolled coagulation, the immune system is continuously exposed to antigens altered by and specifically associated with coagulation. This is analogous to the antibodies generated by exposure to high levels of apoptotic cells generated in animals with deficiencies of clearance, such as complement deficiency and Fas deficiency or in cancer, conditions that are strongly associated with autoimmunity. This hypothesis suggests that the clinical association of FVL and APS is not merely a coincidence, but that chronic coagulation defects combined with external inflammatory stimuli analogous to adjuvant may induce aPL antibodies and also APS. We suggest therefore that chronic coagulation defects should be added to apoptotic-cell-clearance defects, cancer, and infection as significant factors leading to autoimmunity. The hypothesis would explain the linkage of APS with FVL found in a familial study [38]. It would also predict that in humans, the FVL genotype is likely to be associated with higher levels of aPL and perhaps also APS. One report has indeed found increased levels of aPL antibodies in women with FVL taking oral contraception [39]. Another report presented detailed tables of the data from which it was possible to calculate the proportion of APS markers in large groups of patients with FVL compared with normal controls [40]. Using Table 6 of that publication, it is possible to calculate the association of lupus anticoagulant (LA) with FVL both in women with recurrent pregnancy loss and in controls, and this association was highly significant for both groups (χ^2^ test, *P* = 1.4 × 10^-6^, for the combined groups: 26 LA-positive women out of 43 women with FVL, compared with 46 LA-positive women out of 209 controls). By contrast, some investigators have found a reverse relationship between APS and FVL [41]. in a group of women with history of miscarriage and a group of patients with APS, no association was identified between FVL and aPL antibodies [42-44]. There were a number of methodological (selection bias) or molecular mechanisms that may explain this discrepancy. Prospective clinical studies are indicated to definitively examine the prevalence of APS and aPL antibodies in FVL carriers.

## Conclusions

The coagulation-induced autoimmunity hypothesis implies that generation of aPL antibodies in human APS may involve a positive feedback loop, in which procoagulant antibodies generate hypercoagulation, which exposes the immune system to more antigens, consolidating the pathogenic immune response and leading to antigen spread. The therapeutic implication of this hypothesis is that anticoagulant therapy may also have an immunologic effect in APS and contribute to lowering of aPL antibody levels. This hypothesis would also predict that high levels of aPL antibodies would be difficult to induce by β_2_-GPI immunization in β_2_-GPI-deficient mice. In addition, the results of the present study indicate that aPL antibody levels are a significant factor in causing neurodegeneration, and should be targeted directly by therapy.

## Abbreviations

APC: Activated protein C; aPL: Antiphospholipid antibodies; APS: Antiphospholipid syndrome; β2-GPI: β_2_-glycoprotein I; BLS: Bielchowsky staining; CCD: Charge-coupled device; CFA: Complete freund’s adjuvant; CL: Cardiolipin; CNS: Central nervous system; DAPI: 4^′^,6-diamidino-2-phenylindole; eAPS: Experimental antiphospholipid syndrome; ELISA: Enzyme-linked immunosorbent assay; FITC: Fluorescein isothiocyanate; FVL: Factor V leiden; LFB: Luxol fast blue staining; PBS: Phosphate-buffered saline; PVC: Polyvinylchloride

## Competing interests

The authors declare that they have no competing interests.

## Authors’ contributions

AK and JC conducted and helped with the design of all experiments and interpretation of the data, performed the statistical analysis, and participated in the writing of the manuscript. NCG and OT conducted all histological evaluations and contributed to interpretation of results. TE and MB carried out the behavioral and serological studies, respectively. CGP and YS helped with interpretation of the data, and were involved in revising the manuscript. All authors have read and approved the final manuscript.

## Pre-publication history

The pre-publication history for this paper can be accessed here:

http://www.biomedcentral.com/1741-7015/11/92/prepub
